# Hospital-Acquired COVID-19: Case Discussions of Two Patients Treated at a Level I Trauma Center

**DOI:** 10.1155/2021/5531557

**Published:** 2021-08-04

**Authors:** Ryan M. Desrochers, Jonathan D. Gates, Daniel Ricaurte, Jane J. Keating

**Affiliations:** Division of Acute Care Surgery at Hartford Hospital, University of Connecticut School of Medicine, USA

## Abstract

The community spread of COVID-19 is well known and has been rigorously studied since the onset of the pandemic; however, little is known about the risk of transmission to hospitalized patients. Many practices have been adopted by healthcare facilities to protect patients and staff by attempting to mitigate internal spread of the disease; however, these practices are highly variable among institutions, and it is difficult to identify which interventions are both practical and impactful. Our institution, for example, adopted the most rigorous infection control methods in an effort to keep patients and staff as safe as possible throughout the pandemic. This case report details the hospital courses of two trauma patients, both of whom tested negative for the COVID-19 virus multiple times prior to producing positive tests late in their hospital courses. The two patients share many common features including history of psychiatric illness, significant injuries, ICU stays, one-to-one observers, multiple consulting services, and a prolonged hospital course prior to discharge to a rehabilitation facility. Analysis of these hospital courses can help provide a better understanding of potential risk factors for acquisition of a nosocomial COVID-19 infection and insight into which measures may be most effective in preventing future occurrences. This is important to consider not only for COVID-19 but also for future novel infectious diseases.

## 1. Introduction

Since the declaration of the COVID-19 outbreak as a global pandemic in March 2020, the number of positive cases rose rapidly. The role of community spread in the transmission of the virus is well known, and early studies have calculated a mean *R*_0_ for the virus of 3.28, which is 2.5 times as contagious as the typical seasonal flu [1, 2]. Efforts to slow the rate of spread via masks and social distancing have proven effective; however, government implementation of and public compliance with these recommendations remain imperfect [3–7].

Despite this understanding of COVID-19 in the community, little is known about the risk of transmission to hospitalized patients. Although screening stations, visiting limitations, patient and employee testing protocols, and personal protective equipment requirements are implemented on an institutional basis, the efficacy of these measures in preventing nosocomial spread is either poor or poorly understood [8]. While the use of these infection control strategies is widespread and there is evidence to suggest that the risk of nosocomial COVID infection is significantly lower than in the community, single nosocomial cases and even outbreaks within healthcare settings are not infrequent events [9, 10].

Although overall the risk of COVID-19 conversion in our busy, academic, urban, level one trauma hospital was exceptionally low likely due to the robust, practiced, and well-funded anti-infection policies and procedures, this case report discusses two patients that were outliers and therefore are important to discuss. Despite initial admission testing via nasopharyngeal swab declaring each of these patients negative for COVID-19 and serial intermittent testing prior to procedures also resulting as negative, several weeks into their hospitalization they both tested positive prior to transfer to rehabilitation facilities. Based on the timeline in both of these scenarios, these patients are examples of hospital-acquired COVID-19. It is important to review and be aware of these cases in order to better understand potential sources of in-hospital transmission in an effort to identify risk factors and avoid future nosocomial infections.

## 2. Patient 1

**Figure 1:**
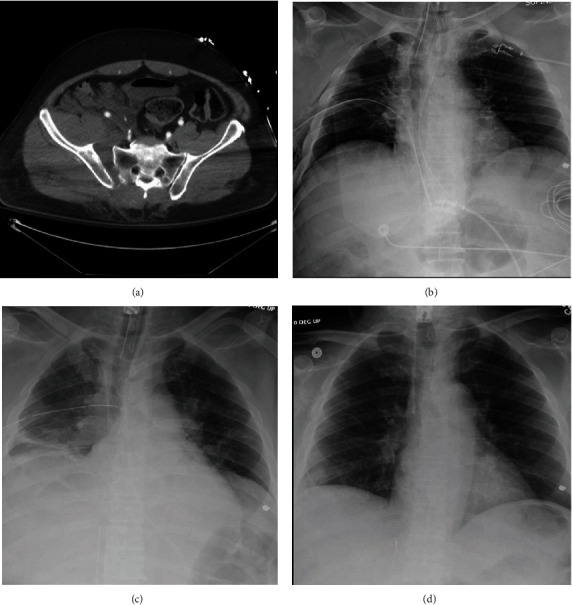
Patient 1 imaging including (a) admission pelvis CT, (b) admission chest X-ray, (c) chest X-ray while on ECMO, and (d) chest X-ray from the time of discharge.

This patient is a 58-year-old man with a history of schizoaffective disorder who presented to the emergency department in October 2020 after he jumped from a third-story window. On arrival to the emergency department, he was noted to have a GCS of 3 with rightward gaze deviation and absent right-sided breath sounds. He was hypoxic and hypotensive with a negative FAST exam. Shortly into his evaluation, he lost palpable pulses and CPR was initiated. An ET tube, femoral cordis, and right chest tube were placed. Return of spontaneous circulation was rapidly achieved, although his blood pressure remained labile. Massive transfusion protocol was initiated for presumed hypovolemic shock. His hemodynamics improved, and he was taken for imaging which revealed an unstable three column fracture of the C7 vertebral body, small bilateral anterior pneumothoraces, a right retroperitoneal hematoma extending along the iliopsoas muscle with active extravasation, and significant osseous injury including displaced bilateral pubic rami fractures, right sacroiliac fracture with joint space widening, a shattered right scapula, and bilateral rib fractures with an underlying right pulmonary contusion (Figure 1). Laboratory evaluation was notable for a negative COVID-19 nucleic amplification assay. He was taken to interventional radiology and underwent embolization of bilateral hypogastric arteries as well as the right L2 and L3 lumbar arteries. Postprocedure, he was taken to the ICU for further management.

In the following days, he was rapidly weaned off of vasopressor support and underwent multiple operations with orthopedic surgery for external fixation of his pelvis and anterior fusion of his C7 fracture. During this time, he became increasingly hypoxic and required additional ventilator support secondary to his pulmonary contusions, numerous rib fractures, and net positive fluid status, eventually requiring transition to venous-venous extracorporeal membrane oxygenation on hospital day 3. He was managed supportively and diuresed as his ECMO requirements weaned. He was found to have an Enterobacter/Streptococcus pneumoniae pneumonia and was treated with Levaquin as per infectious disease recommendations. He was eventually removed from ECMO on hospital day 7.

With his improving condition, he was stable enough to undergo IVC filter placement on hospital day 8. In preparation for this procedure, he had a repeat COVID-19 nucleic amplification assay which resulted negative. Plans were made for permanent fixation of his pelvis at this point as well, but were continually delayed secondary to fevers and agitation. Psychiatry input was obtained in management of his medications. His pelvis was internally fixated on hospital day 20 while the external fixation device remained in place per orthopedics recommendations, and he was able to be extubated the following day. On hospital day 22, he was able to be more formally evaluated by the psychiatry team, who recommended a one-to-one sitter at all times given his agitation and poor mental status. He was able to be transferred out of the ICU on hospital day 24, after which he passed a swallow evaluation and was started on a diet, began aggressive physical therapy, and had his medications titrated by the psychiatry team. He was briefly set back by an ileus and colonic pseudo-obstruction, which were able to be managed conservatively. He was transferred to floor level of care on hospital day 32.

On hospital day 34, he was noted to have bilateral lower extremity deep vein thromboses and was started on therapeutic doses of Lovenox. In preparation for discharge, his sister was named his conservator of person and estate. In addition, plans were made for removal of his external pelvis fixation device by the orthopedics team. Preoperative COVID nucleic amplification assay on hospital day 41 resulted positive. The patient was asymptomatic at this time, and the case proceeded as planned on hospital day 42. At this point, he was allowed to be weight bearing as tolerated to his bilateral lower extremities, and he continued his physical therapy. Given his now known COVID-positive status, he was placed on contact, droplet, and airborne precautions and given an isolated room in a low-traffic section near the back of the wing given a paucity of negative pressure rooms. As he was readied for discharge, he remained asymptomatic and no COVID treatment was initiated as per infectious disease recommendations. He was taken off of these COVID precautions on hospital day 52, 10 days after his positive test, as per institutional policy. He was ultimately discharged to a rehab facility on hospital day 57 without repeat COVID testing.

## 3. Patient 2

**Figure 2:**
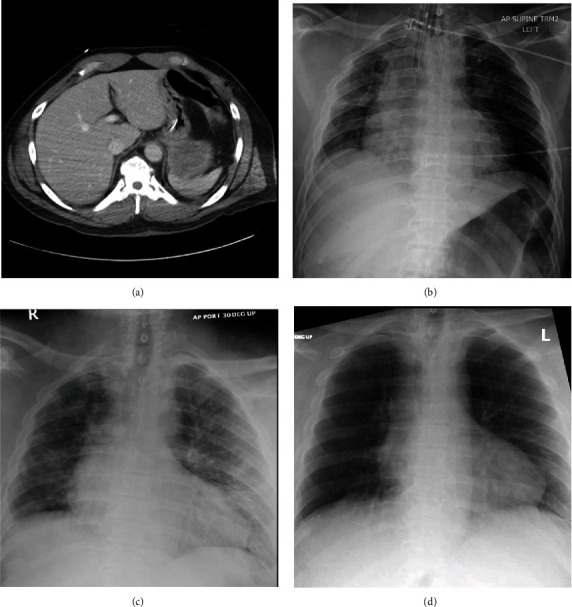
Patient 2 imaging including (a) admission abdominal CT with air pattern concerning for diaphragmatic injury, (b) admission chest X-ray, (c) chest X-ray after positive COVID test, and (d) chest X-ray from the time of discharge.

This patient is a 54-year-old homeless man with a history of schizophrenia who presented to the emergency department in November 2020 after being found down with a penetrating injury to the left anterior chest. On arrival, his GCS was 3, and he was being ventilated via bag valve mask by EMS. He was promptly intubated for airway protection. His vital signs were stable. On further exposure, he had an approximately 3 cm stab wound to his left anterior chest, medial to the nipple line, at approximately the level of the 7^th^ rib. Otherwise, he was noted to have ecchymosis of the occiput and superficial lacerations to his right flank. Also concerning was a fixed and dilated left pupil. A pan CT scan revealed a left subdural hematoma with bilateral frontal hemorrhagic contusions and 5 mm of midline shift. Additionally, he was found to have a moderate left-sided pneumothorax and associated left anterior 7^th^ rib fracture for which he underwent chest tube placement with resolution of his pneumothorax (Figure 2). A cerebral oxygenation monitor and drainage device was placed by neurosurgery revealing an elevated opening pressure of 31 mmHg. At this time, there was also concern for possible diaphragmatic injury given the location of his stab wound and imaging findings; however, in the setting of his elevated intracranial pressures, he was deemed unsafe for the OR and admitted to the neuro ICU for further monitoring.

On hospital day 0, his routine workup included a negative COVID-19 nucleic amplification assay. His intracranial pressure was controlled using mannitol and hypertonic saline bullets while he was started on levetiracetam for seizure prophylaxis. Repeat CT imaging the following day showed stability in his intracranial bleeds and a decrease in midline shift. His mental status improved, and he self-extubated on hospital day 3. After his intracranial pressures normalized his drains were removed, and on hospital day 6, he underwent diagnostic laparoscopy to rule out diaphragmatic injury given the location of his initial stab wound. Prior to this procedure, he had a repeat COVID nucleic amplification assay as per institutional policy, which again resulted negative.

Following this intervention, his condition continued to improve. His diet was advanced as tolerated and he was otherwise normalized, although he continued to have issues with agitation and altered mental status secondary to his traumatic brain injury and underlying schizophrenia. Psychiatry was consulted for titration of his medications, and plans were made for discharge to a TBI rehab; however, his discharge was complicated by his homelessness status and frequent behavioral outbursts, occasionally requiring chemical sedation. Beginning on hospital day 13, he was given a one-to-one bedside nursing assistant and was occasionally placed in a SOMA Safe Enclosure bed.

In anticipation of discharge, repeat COVID nucleic amplification assay was done in compliance with rehabilitation center policies. Tests sent on hospital days 49 and 50 both resulted negative; however, an additional test sent on hospital day 55 resulted positive. As before, the patient was given an isolated room and placed on contact, droplet, and airborne precautions. Later that evening, the patient became febrile to 103°F but refused additional fever workup. A second COVID nucleic amplification assay was repeated the following day, which confirmed his COVID-positive status. He was treated with a 10-day course of dexamethasone as per infectious disease recommendations, and his fevers resolved. Repeat COVID testing on hospital day 66 remained positive, and he had his first negative test on hospital day 72. Shortly thereafter, he was accepted to a facility for ongoing rehabilitation.

## 4. Discussion and Conclusions

These two complex trauma patients, admitted in October and November of 2020, required a multidisciplinary effort at our level one trauma center in order to treat their myriad of injuries. Likewise, they both suffered from psychosocial issues which at least in part delayed their discharge from the hospital to a rehabilitation facility. Both patients underwent multiple procedures, were seen by several consultants, and at least for a portion of their stay required a one-to-one bedside monitor by a nursing assistant for their safety and impulsiveness. These factors certainly increased their exposure to hospital personnel which in turn may have increased the chance of infection given the virus's mode of transmission.

For the duration in which both of these patients were admitted, it was hospital policy that all employees and visitors entering the building undergo mandated temperature, symptom, and risk factor screening via thermal imaging system and brief questionnaire regarding recent symptoms or travel. Masks were required on campus at all times. Employees and visitors were required to undergo an at-home quarantine following any known COVID-19 exposure or travel greater than 24 hours to states with a higher incidence unless proof of a negative COVID test since the event was provided. Universal precautions for patient interactions included a three-layer surgical mask, which were provided by the hospital for employees, and all patients received a COVID test prior to admission. Patients with unknown COVID status were often treated as COVID positive, and employees were encouraged to wear an N95 and face shield during these interactions. In addition, hospital visitation was limited to one visitor per patient at any given time, and restrictions were placed on the duration of visitation, with case-by-case exceptions made for family meetings and patients receiving end-of-life care. COVID testing was available upon immediate request of employees, and incidences of outbreaks among staff members were investigated with required testing for those deemed exposed by contact tracing.

Additionally, one of our postoperative patients had symptomatic community-acquired COVID-19 resulting in fever. Fevers postinjury and postoperatively are common, and the surgical services are well versed in the identification and management of those conditions. Throughout the pandemic, our practice included adding a sixth W to the five “W's” of postoperative fever: that of “W(ariness) for COVID-19.” In the midst of this pandemic, postoperative fever workup thus included COVID-19 testing. Certainly, the combination of fever and new infiltrates on chest film or chest CT should elevate the concern. Additionally, deep venous thrombosis (DVT) is a recognized complication from injury and surgical intervention. The incidence in the hospitalized COVID-19 patients has been reported to be approximately 17% [11, 12]. It is difficult to know whether patient 1 developed a DVT from the sequelae of his injury alone or whether the transition to COVID-positive status may have contributed to a worsened hypercoagulable state.

In these patients, it is difficult to discern what changes could be made to have prevented their acquisition of COVID-19. Ideally, it would have been best to minimize in-person contact as this is likely the biggest risk factor contributing to their contracture of the virus; however, the care of these patients required the use of a one-to-one sitter and multiple consulting services in order to maintain patient safety and deliver appropriate care. To our knowledge, neither of these cases involved a breach of universal precaution or known exposure to a COVID-19 positive individual, whether that be staff or visitor. At a minimum, these cases underscore the need to maintain vigilance, never let down one's guard, and consider further workup of the postinjury and postoperative fever to include continued COVID-19 swabs despite prior negative studies during the same hospitalization. It is important to discuss and keep track of these patients in order to identify risk factors for COVID conversion going forward. Furthermore, in order to keep hospital staff safe from infection, it may be necessary to schedule routine COVID-19 testing intervals in order to detect nosocomial spread. This discussion applies not only to COVID-19 but also to future infectious disease outbreaks.
